# Occupancy Grid Mapping in Urban Environments from a Moving On-Board Stereo-Vision System

**DOI:** 10.3390/s140610454

**Published:** 2014-06-13

**Authors:** You Li, Yassine Ruichek

**Affiliations:** IRTES-SET, Université de Technologie de Belfort-Montbéliard, Belfort Cedex 90010, France; E-Mail: yassine.ruichek@utbm.fr

**Keywords:** occupancy grid map, U-V disparity image, intelligent vehicles

## Abstract

Occupancy grid map is a popular tool for representing the surrounding environments of mobile robots/intelligent vehicles. Its applications can be dated back to the 1980s, when researchers utilized sonar or LiDAR to illustrate environments by occupancy grids. However, in the literature, research on vision-based occupancy grid mapping is scant. Furthermore, when moving in a real dynamic world, traditional occupancy grid mapping is required not only with the ability to detect occupied areas, but also with the capability to understand dynamic environments. The paper addresses this issue by presenting a stereo-vision-based framework to create a dynamic occupancy grid map, which is applied in an intelligent vehicle driving in an urban scenario. Besides representing the surroundings as occupancy grids, dynamic occupancy grid mapping could provide the motion information of the grids. The proposed framework consists of two components. The first is motion estimation for the moving vehicle itself and independent moving objects. The second is dynamic occupancy grid mapping, which is based on the estimated motion information and the dense disparity map. The main benefit of the proposed framework is the ability of mapping occupied areas and moving objects at the same time. This is very practical in real applications. The proposed method is evaluated using real data acquired by our intelligent vehicle platform “SeTCar” in urban environments.

## 1. Introduction

In the field of intelligent vehicles, many tasks, such as localization, collision avoidance and path planning, are usually performed based on well-represented maps [1,2]. The occupancy grid map (OGP) [3,4] is one of the most popular environmental representation tools. It maps the environment around a vehicle as a field of uniformly-distributed binary/ternary variables indicating the status of cells (occupied, free or undetected). Besides, as a practical instrument for environmental understanding, the occupancy grid map is very useful for integrating different sensor measurements (radar, LiDAR, vision system) into a unified representation.

In the literature, range sensors, such as LiDAR and radar, are usually used for creating occupancy grid maps. The characteristic of measuring distance directly makes occupancy grid mapping easily performed. Usually, under a given sensor measurement model (such as the inverse sensor model [4]), probabilistic occupancy grid mapping is able to be quickly calculated with the measurements. However, in contrast to pervasive applications of visual systems in intelligent vehicles, occupancy grid mapping by visual systems is not well researched. In addition, in previous research [3,4], occupancy grid mapping is served by a static environment. After the map is generated, it is stored for future usage, whereas the situation changes in applications of intelligent vehicles. In our applications, an intelligent vehicle has to drive by itself in a dynamic, unknown urban area. Therefore, the subsequent change emphasizes the ability of mapping in dynamic environments in real time without prior information.

**Figure 1.**
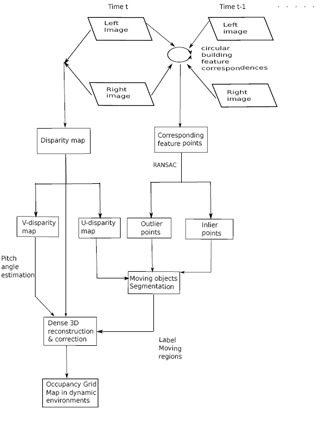
The flow diagram of the proposed method.

This paper proposes a framework of stereo-vision-based dynamic occupancy grid mapping in urban environments. The dynamic occupancy grid map models real environments by evenly distributed rectangle grids, which contain both occupancy and motion information. The proposed framework mainly comprises two components (motion analysis for the vehicle itself and independent moving objects and dynamic occupancy grid mapping) within two parallel processes (sparse feature points processing between two consecutive stereo image pairs and dense stereo processing). Figure 1 visualizes the whole framework. For every incoming stereo image pair, sparse image feature points are extracted and tracked in a circular manner [5] between the current and previous image pairs. The successfully tracked feature points are used to estimate the ego-motion of the vehicle itself and the independent motions of the surrounding moving objects. Meanwhile, dense stereo disparity is calculated from each stereo image pair. A pixel-wise moving object segmentation is performed in a U-disparity map. Finally, the dense stereo information, together with the moving information, is used to create a dynamic occupancy grid map. This paper is extended from our previous publication [6] in adding a comparison of image features within our application background and more detailed descriptions and experiments. The contributions of this paper are:

- A detailed comparison of different kinds of image feature points in the application of motion estimation. In this paper, we evaluate and compare at length the performances of various kinds of image feature point-in-motion estimation, as well as the advantages and disadvantages of feature matching and tracking.
- A novel independent moving object segmentation method based on a U-disparity map. From sparse independent moving feature points, we utilize the U-disparity map to achieve pixel-wise moving object segmentation in a disparity map.
- Improved dynamic occupancy grid mapping method. Based on previous work in [6], we propose a dynamic occupancy grid mapping method with consideration for the pitch angle between the stereo-vision system and the ground plane.

This paper is organized as follows. Section 2 reviews several research works in motion analysis from a moving platform and visual-based occupancy grid mapping. In Section 3, we explain the foundations of the sensor model for our stereoscopic system and the definition of the dynamic occupancy grid map. Approaches to compute the disparity map and its variations, U-V disparity maps, are described in Section 4. In Section 5, a sparse feature point-based motion analyzing method and an independent moving objects segmentation method in the disparity domain are presented. Section 6 presents a probabilistic occupancy grid mapping technique representing both obstacles and moving objects. Section 7 shows experimental results with real data sets. Section 8 summaries this paper and provides prospects for future improvements.

## 2. Related Works

### 2.1. Motion Analysis from a Moving Vision System

Motion analysis from a moving platform consists of ego-motion estimation and independent moving estimation. Ego-motion estimation from a moving vision system is also coined as visual odometry. Here, we only review stereo-based visual odometry.

Visual odometry from stereo images was initialized by Moravec [7] and later developed by Matthies [8]. Consequent refinements were mainly focused on establishing accurate and robust pairwise correspondences, such as [9] (introducing RANSAC [10]) and [11] (introducing sliding window bundle adjustment). Improvements have been developed also around different motion estimation mechanisms, such as [12] (using 3D-3D point registration) and [13] (using a 2D-2D quadrifocal constraint). Improvements have continued until the successful real-time application in the Mars Exploration Rover [14], which is a milestone for the success of visual odometry. In recent years, [5] has contributed to an open source visual odometry library, LibVISO2. To sum up, a general procedure for stereo visual odometry can be roughly summarized as: first, establishing point-to-point correspondences through subsequent stereo images; second, estimating ego-motion from 2D-2D [13] 3D-3D [12] or 3D-2D [5] point constraints.

As for the independent detection of a moving object or the segmentation from moving vision systems, this is a classic, but still open research area. The proposed approaches could be roughly divided into two categories. The first one uses global motion compensation to generate a background model as utilized in motion detection in static camera cases [15]. This method suffers from severe limitations in the assumption of the homography transform between consecutive images. Although several improvements have been introduced in [16,17], it is still unable to deal with complex environments. The second category of approaches is based on analyzing displacements of pixels in the image plane (optical flow) [18,19] or in the 3D real world (scene flow) [20,21]. This kind of method usually involves the joint estimation of ego-motion, as well as object movement. The benefits of the second category are no assumptions for specific environments and the ability to estimate motions at the same time. The proposed method in this paper belongs to the second category. It is able to easily be integrated into the framework of visual odometry. Due to the sparse nature of the proposed method, it is not stable enough in comparison to dense approaches.

### 2.2. Vision-Based Occupancy Grid Mapping

In [22], the authors regard a stereo sensor as a range finder, taking the first encountered object in each column as an occupied cell. Braillon *et al.* [23] firstly estimated the ground plane in front of a stereo camera, then clustered the detected points above the plane as occupied cells. Three different types of occupancy grid maps are analyzed and compared at length in [24], which furthermore proposes three kinds of occupancy likelihood functions modeled by Gaussian distribution. Quite similar to [24], the method proposed in [2] introduces an inverse sensor model for a stereo camera. In [25], the occupancy grid map is generated from a digital elevation map after filtering out road and isle cells according to height information.

In [26], the authors directly calculate occupancy grids by several effective probabilistic occupancy grid models in an obstacle U-disparity image. In addition, this method requires a pre-performed road-obstacle separation. Compared to the existing methods, the main differences of the proposed framework lie in: firstly, we improve the occupancy grid mapping results by ground plane analysis based on the V-disparity image. Secondly, we extend the original occupancy grid mapping scheme to a dynamic occupancy grid mapping framework, which is able to label the moving objects in the local map.

## 3. Basics

### 3.1. Platform and Sensor Model

**Figure 2.**
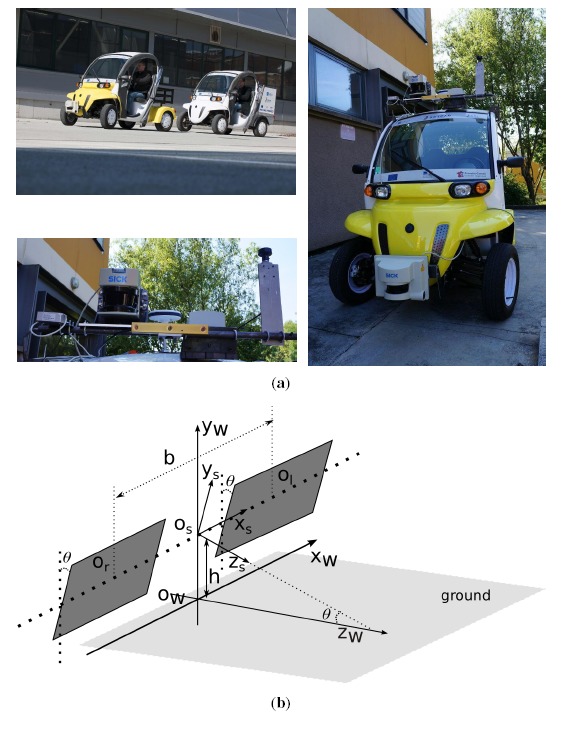
The platform and geometric model. (**a**) Our platform, SetCar, and the equipped sensors; (**b**) geometric model of the stereo vision system.

Our platform, the SetCar, in UTBMis an electric vehicle equipped with multiple sensors, such as a laser range finder, a stereo vision system, a fish-eye camera, GPS, IMU, *etc*, as shown in Figure 2a. This platform is developed as a prototype of an intelligent vehicle aimed at being capable of autonomously driving in urban environments.

In our platform, a binocular stereo vision sensor (Bumblebee XB3) (seen in the lower picture of Figure 2a) is mounted on top of the vehicle. The stereoscopic system was previously calibrated and rectified by [27]. Therefore, the left and right cameras are viewed as the same and modeled by the classic pinhole model (*f, c_u_, c_v_*), where *f* is the focal length, (*c_u_, c_v_*) is the position of the principal point. Furthermore, the baseline length *b* is calculated from extrinsic stereo calibration. As shown in Figure 2b, the ground is assumed to be a flat plane under the stereo vision system. The stereoscopic coordinate system is assumed to be originated from *O_s_*, the middle point of the baseline. The world coordinate system is set as originated from the point *O_w_*, the projection of *O_s_* in the ground. The left and right camera frames are assumed to have the same pitch angle *θ* with regard to the ground plane. x-y-z directions are illustrated, as well, in Figure 2b. Therefore, the 3D position of a point (*X_s_, Y_s_, Z_s_*) in the stereoscopic coordinate system can be triangulated from its projections (*u_l_, v_l_*) and (*u_r_, v_r_*) in the left and right image planes as:

(1) $$\left[\begin{array}{c} X_{s}\\ Y_{s}\\ Z_{s} \end{array}\right]=\left[\begin{array}{c} (u_{l}-c_{u})\cdot b/\Delta -b/2\\ (\upsilon_{l}-c_{\upsilon })\cdot b/\Delta \\ b\cdot f/\Delta \end{array}\right]$$

where Δ is the disparity. Since there is a pitch angle from the stereoscopic system to the ground, the corresponding coordinate in the world coordinate system has to be corrected as:

(2) $$\left[\begin{array}{c} X_{w}\\ Y_{w}\\ Z_{w} \end{array}\right]=\left[\begin{array}{ccc} 1 & 0 & 0\\ 0 & \text{cos}\theta & -\text{sin}\theta \\ 0 & \text{sin}\theta & \text{cos}\theta \end{array}\right]\;\left[\begin{array}{c} X_{s}\\ Y_{s}\\ Z_{s} \end{array}\right]+\left[\begin{array}{c} 0\\ h\\ 0 \end{array}\right]$$

where *θ* is the pitch angle and *h* is the height between the stereoscopic system and the ground plane.

### 3.2. Definition of Dynamic Occupancy Grid Map

A dynamic occupancy grid map *ℳ* is defined as an evenly distributed rectangular array with predefined cover area 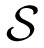. The size of each cell *C_i,j_* in the map is also set as 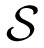
_*C*_, where *i* and *j* are indices of a cell in the grid map. Every cell *C_i,j_* holds a two-dimensional state vector [*O_i,j_, M_i,j_*], where *O_i,j_* is a ternary occupancy indicator consisting of three states, and *M_i,j_* is a binary moving indicator comprising two states:

(3) $$O_{i,j}=\{\begin{array}{l} \text{undetected}\\ \text{occupied}\\ \text{free} \end{array}\;\text{and}\;M_{i,j}=\{\begin{array}{l} \text{dynamic}\\ \text{static} \end{array}$$

## 4. Creating Disparity Map and U-V Disparity Maps

**Figure 3.**
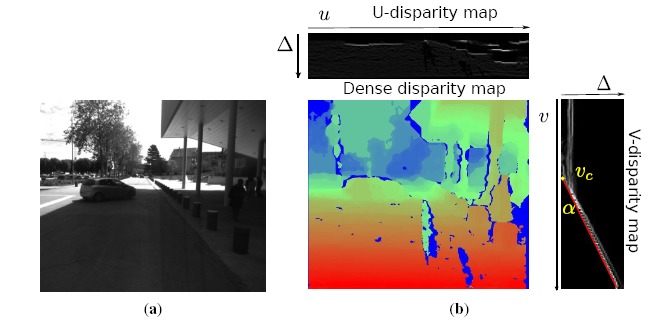
Stereo measurements. (**a**) An image of a road scene; (**b**) the dense disparity map and U-V disparity maps.

In parallel with sparse feature processing, a dense stereo vision processing is performed for further independent moving object segmentation and dynamic occupancy grid mapping. Because of the good performances in both accuracy and speed, the semi-global block matching (SGBM) algorithm [28] is employed to compute dense disparity image *I*_Δ_. Figure 3b shows a disparity image calculated from Figure 3a by the SGBM algorithm.

Furthermore, U-V disparity maps ([29–31]) are calculated for scene understanding. In the field of intelligent vehicles, U-V disparity images are helpful and practical tools for the aim of scene understanding (obstacle detection, ground plane detection, *etc.*). As a transformation of the dense disparity image, U-V disparity images are generated by accumulatively projecting dense disparity images into the rows/columns. Specifically, the U-disparity image is built by accumulating the pixels with the same disparity in *I*_Δ_ in each column (*u*-axis). Hence, the intensity *I*_Δ_ means that there are *I*_Δ_ pixels in the *u*-th column that have the same disparity value Δ. The V-disparity image is calculated symmetrically. Examples are shown in Figure 3b. Actually, the U-disparity image could be viewed as a bird's view disparity image of the scene, while in the V-disparity image, the ground plane is mapped to a quasi-line (marked as a red line in Figure 3b). In our framework, the U-disparity image will be used for moving object detection and segmentation, while the V-disparity image will be used for estimating the ground pitch angle and then correcting the 3D reconstruction.

## 5. Motion Analysis from Moving Stereo Vision Platform

Motion estimation from sparse feature points usually demands establishing point-to-point correspondences between extracted feature points in multiple images. In this section, we firstly review several kinds of image feature detectors and approaches to establish such point-to-point correspondences. Next, we introduce a method of ego-motion estimation. At last, independent moving objects are detected and segmented based on the U-disparity map.

### 5.1. Ego-Motion Estimation

#### 5.1.1. Feature Points Detection

In the literature of computer vision, interest point detection is aimed at extracting interest points or image patterns that differ from their neighborhoods.

- Corner detectors hold a large part of the interest point detectors, such as the famous Harris corner detector [32], which derives a “corner strength” from a second-order moment image gradient matrix. Shi and Tomasi [33] developed this method in the “Good Feature To Track” (GFTT) by using the smallest eigenvalue of the auto-correlation matrix as the corner strength function. The GFTT is proven to be stable in feature point optical flow tracking. The FAST detector [34] considerably speeds up the detection time by comparing pixels on a circle of fixed radius around the potential feature point.
- As for blob detectors, the famous SIFT detector [35] achieves scale and rotation invariance by extracting the local extrema of an image filtered with differences of Gaussians (DoG). Similar to SIFT, the SURFdetector [36] uses a fast Hessian to improve the detection speed. The SURF detector is also invariable to scale and rotation change to some extent. The STAR [37] introduces a suite of scale-invariant center-surround detectors to improve stability and accuracy.

s For more discussion about feature point detectors, a comprehensive overview can be found in [38]. Generally speaking, corner-like detectors (Harris, GFTT, FAST) are fast to compute, but less distinctive, whereas blob detectors (SIFT, SURF, STAR) are more distinctive in scale and affine change, but slow in detection. In addition, corner-like detectors usually perform more accurate localization. Hence, selecting a suitable feature detector should depend on the requirements of real applications.

In our framework, we choose STAR as the feature detector after an overall consideration. An evaluation of various feature detectors in real urban environments is given in Section 7.

#### 5.1.2. Establishing Point-to-Point Correspondence

After extracting sparse interest feature points, there are two strategies to build point-to-point correspondences between those points through subsequent frames. The first one tracks detected features in all of the candidate images. This approach is appropriate when appearance changes between subsequent frames are small, which means that the subsequent images are acquired at nearby positions. One typical method is the “Kanade–Lucas–Tomasi” (KLT) feature tracker [33].

An alternative to tracking features is to detect feature points in all candidate images, next extracting a compact feature descriptor for each feature point and, then, finding corresponding features by matching the feature descriptors. Although the sum of squared differences (SSD) or the normalized cross-correlation (NCC) around a feature point can be utilized as feature descriptors for feature matching, they are not invariant to the changes of scale, viewpoint and orientation. One of the most popular descriptors is SIFT [35], which is a 128-element descriptor vector about the histogram of local gradient orientations. SIFT is capable of being against changes in illumination, rotation and scale to a certain degree. However, its computing speed is slow. SURF [36] is proposed as a faster alternative to SIFT, by adopting efficient box filters instead of the computationally expensive Gaussian filters. In recent years, several binary descriptors, BRIEF [39], ORB [40], BRISK [41] and FREAK [42], have attracted much attention for high computation speeds and prospects in mobile applications. Those methods use pairwise intensity comparisons in a patch around a keypoint to achieve rapid calculation.

**Figure 4.**
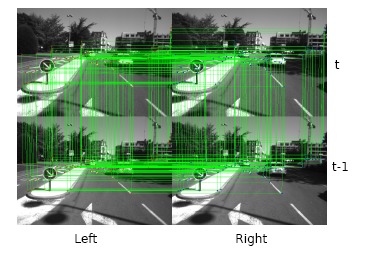
Circular feature tracking between two subsequent stereo image pairs after a refine process.

In the proposed framework, we choose a KLT feature tracker to establish feature point correspondences. The STAR feature points are detected in the current left image and, then, tracked in a circular manner (a point tracker starts from the left image in time *t*, across the right image in time *t* and *t* – 1, reaches the left image in time *t* – 1 and, finally, back to the starting image). The circular tracking process is shown in Figure 4. A refining process is applied after the tracking procedure to filter out some obvious inaccurate trackers. The advantages and disadvantages about feature tracking and matching are discussed at length in Section 7.

#### 5.1.3. Motion Calculation

After the above two steps, sparse feature points are extracted, and correspondences are established through four images [5], which are the left and right images acquired at two consecutive times. Hence, for each consecutive stereo image pair, we have a tuple of feature points as: $\left\{(u_{l}^{t},\upsilon_{l}^{t})↔(u_{r}^{t},\upsilon_{r}^{t})↔(u_{l}^{t-1},\upsilon_{l}^{t-1})↔(u_{r}^{t-1},\upsilon_{r}^{t-1})\right\}$, where $\left(u_{l}^{t},\upsilon_{l}^{t}\right)$ and $\left(u_{r}^{t},\upsilon_{r}^{t}\right)$ are feature points in the left and right images captured at time *t*, respectively. The 3D positions of the selected feature points are calculated by Equation (1). Due to the well-known big errors of point triangulation at long distance, we set a region of interest (ROI) and filter out matched feature points far away from the stereo camera.

Here, the 3D-2D constraint [5] is utilized to estimate ego-motion. Let the left image frame be the reference coordinate system. Assuming the motions of all corresponding feature points are caused by ego-motion, the motion parameters (rotation/translation parameters), the 3D positions of a feature point *P*^*t*−1^ in time *t* − 1 and its image coordinates *p^t^* at time *t* are related by:

(4) $${\tilde{p}}^{t}=\text{P}\cdot (\;\left[{\text{R}}^{t-1}|{\text{T}}^{t-1}\right]\cdot {\tilde{P}}^{t-1})$$

where the notation ·̃ denotes homogeneous coordinates, **P** denotes the 3×4 projection matrix of the left camera and **R**^*t*−1^ and **T**^*t*−1^ are the movement parameters of the left camera within the interval [*t*−1 : *t*]. Let ( $P_{i}^{t}$, *i* = 1,…, *N*) denote the 3D positions of matched feature points within an ROI at time *t*. The ego-motion parameters are estimated by the Gaussian–Newton method to achieve minimization:

(5) $$\min{\sum_{i=1}^{N}\left\|\text{P}\cdot ({\tilde{P}}_{i}^{t}-[{\text{R}}^{t-1}|{\text{T}}^{t-1}]\cdot {\tilde{P}}_{i}^{t-1})\right\|}^{2}$$

**Figure 5.**
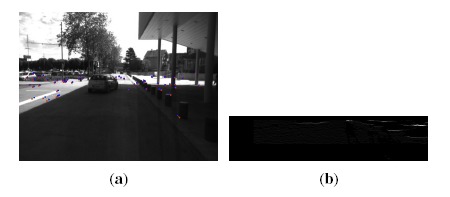

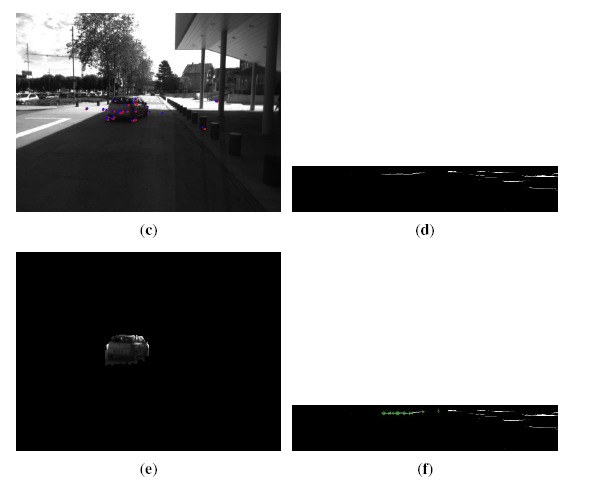
Motion analyzing from feature correspondences and the U-disparity image. (**a**) Inlier feature points after grouping; (**b**) corresponding U-disparity image; (**c**) outlier feature points after grouping; (**d**) the U-disparity image after intensity adjustment; (**e**) moving object segmentation; (**f**) outliers projected into the U-disparity map.

However, in real urban environments, many movements of corresponding feature points are caused by independent moving objects or tracking/matching noises. A robust statistic method, RANSAC, in cooperation with the Gaussian–Newton method, is used to identify the inliers and outliers and, meanwhile, to estimate the ego-motion parameters. The inliers are points following the movement of our experimental platform, while the outliers are points consisting of independent moving objects and noises. The detected inliers/outliers are shown in Figure 5a,c (Detecting ROI is set to a maximum of 20 m in distance and a maximum of 3 m in height).

### 5.2. Independent Moving Objects Segmentation in the U-Disparity Map

As introduced in Section 4, the U-disparity map is a projection of pixels in the dense disparity map along the columns. The intensity of a pixel in U-disparity map *I_u_*(*u*,Δ) represents the number of pixels with the same disparity Δ in column *u* in the dense disparity map. Since in most cases in urban environments, an intelligent vehicle drives on a flat ground plane with a certain pitch angle, the intensity value of a pixel in the U-disparity map indicates whether it belongs to an obstacle or not. Furthermore, one of the most important attributes of U-disparity used for obstacle segmentation is that, = an obstacle on the ground is projected as a “bright' line in U-disparity, as shown in Figure 5b. This attribute of the U-disparity image provides an efficient way to detect or segment obstacles in urban environments.

In the proposed framework, independent moving object segmentation in a dense disparity image is achieved based on U-disparity image segmentation. Noticing that the “outlier” feature points as by-products in ego-motion estimation originate from independent moving objects or noises. When projecting these outliers into the U-disparity image, they are always located in the “bright line” in the U-disparity map. Therefore, it is intuitive to take those outliers as seeds and use a flood-fill [43] segmentation method to segment independent moving objects in the U-disparity map.

**Figure 6.**
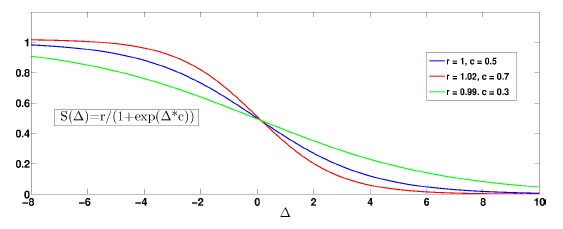
Modified sigmoid functions for intensity adjustment.

However, before performing flood-fill segmentation in the U-disparity map, an issue should be correctly addressed. In practice, an object close to the stereo vision system would be always captured in more pixels than an object far from the system. This means that in the U-disparity map, the objects with large disparities are always “brighter” than the objects with small disparities, as in Figure 5b. This phenomenon would cause segmentation faults. To even out the gray value distribution in the U-disparity map, we employ an intensity adjustment. A modified sigmoid logistic function 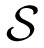
(·) [44] is used to adjust the intensity of the U-disparity map:

(6) $$I_{u}^{\prime }=I_{u}\cdot \text{S}(\Delta )=I_{u}\cdot \frac{r}{1+e^{\Delta \cdot c}}$$

where *I_u_* and $I_{u}^{\prime }$ are the intensities of a pixel in the U-disparity map before and after adjustment, respectively. *r* and *c* represent control coefficients. Δ is the row in the U-disparity map. The sigmoid function is able to smoothly restrain the intensity of areas near the stereo vision system and to amplify the intensity of the areas far away. Noticing that Δ is bigger when an object is closer, an illustrative example is in Figure 6, where three modified sigmoid functions with different parameters are given. With tuned parameters, an example of the U-disparity map after intensity adjustment is shown in Figure 5d. We can see that the intensities of all potential objects are adjusted similarly to each other, regardless of the distance.

Based on the corresponding feature points and adjusted U-disparity map, we segment the independent moving objects in a stereo image pair acquired at time *t* as follows:

1. Project all of the outliers into the adjusted U-disparity map according to their disparities, as shown in Figure 5f.
2. Take the new locations of outliers in the adjusted U-disparity map as seeds. Then, a flood fill segmentation algorithm is performed to segment image patches with similar intensities to the seeds. All of the candidate segments are stored for further refinement.
3. After obtaining all of the candidate segmentation patches, a merging process merges all of the segments that are mutually overlapped.
4. Since the outliers comprise inaccurate tracked feature points appearing in static obstacles or noises, incorrect segments would lie in candidate segments. To overcome this problem, a double-phase post-processing refinement is applied. In the first phase, if any candidate segment contains an inlier projection in the U-disparity map, it is rejected. In the second phase, the surviving segments are compared to stored candidate segments in previous time *t* − 1. If a segment has an overlapped region with a previous segment, it passes the refinement and is confirmed as an independent moving object in U-disparity. The left candidate segments are stored for usage in the next frame.
5. At last, confirmed segments in the U-disparity map are back projected to the dense disparity map to get independent moving objects in the dense disparity map. An example is shown in Figure 5e.

## 6. Building Dynamic Occupancy Grid Map

To build a dynamic occupancy grid map, a dense 3D point cloud is triangulated from the stereo image pair by Equation (1) and corrected by the pitch angle of the stereo vision system at first. The reconstructed 3D points are within the coordinate system of stereoscopic system *O_s_*, as shown in Figure 2 and described in Section 3.1. Then, the reconstructed 3D points are assigned to each cell with a predefined resolution. Noticing the assumption that all obstacles are perpendicular to the planar ground, the greater the number of points a cell holds, the greater the probability of it being occupied. Similarly, the higher the average height of the points a cell holds, the more probable it to be occupied. Consequently, we separately compute the occupancy probabilities *P*(*O*∣*num*) and *P*(*O*∣*height*) for each grid. Then, we take the weighted average of the two values as the final occupancy probability. A motion indicator *M* of a cell is decided by counting the numbers of 3D points from independent moving segments.

### 6.1. Preprocessing: Correcting 3D Points

Most of the existing stereo-based occupancy grid mapping methods [2,24,26] assume that the stereoscopic system is parallel to the ground. In our work, we rectify reconstructed 3D measurements by estimating the pitch angle between the stereoscopic system and ground plane. We will show that this correction improves the quality of the occupancy grid map.

One attribute of V-disparity is that the ground plane is projected into a line, as the red line drawn in Figure 3b. Let the equation of the ground's projection in the V-disparity plane be: *V* = *α*Δ + *υ_c_*, where *α* is the slope, Δ is the disparity and *υ_c_* is the value when Δ = 0. It can be inferred that a plane with equation *Z* = *aY* + *d* in the world coordinate system is projected in V-disparity as [30]:

(7) $$\Delta =\frac{b}{\alpha h-d}(\upsilon -c_{\upsilon })(a\sin\theta +\cos\theta )+\frac{b}{\alpha h-d}f(a\cos\theta -\sin\theta )$$

where *h, θ* are the height of the camera coordinate system to the ground plane and pitch angle, respectively. For planar ground, the pitch angle can be deduced as [30]:

(8) $$\theta =\text{arctan}(\frac{c_{\upsilon }-\upsilon_{c}}{f})$$

**Figure 7.**
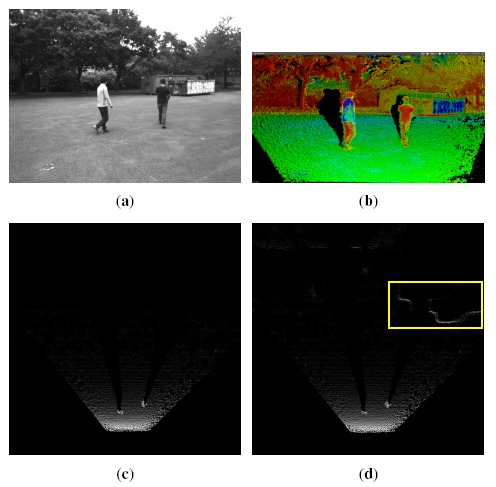
The effect of pitch angle correction. (**a**) A scene consisting of two pedestrians and obstacles; (**b**) 3D reconstruction from the disparity image; (**c**) projection of all of the 3D points to the ground before pitch angle correction; (**d**) projection of all of the 3D points to the ground after pitch angle correction.

In real environments, the ground plane is projected to a quasi-line in the V-disparity image. Hence, we use the Hough transform to extract this line and calculate the pitch angle by Equation (8). After estimating the pitch angle, all of the reconstructed points acquired by triangulation are corrected according to Equation (2). The benefit of correction is illustrated in Figure 7a,b. Figure 7a is a bird's view of the reconstructed 3D point cloud of Figure 3a before correction. The more points within one area, the higher intensity it has. From Figure 7b, the result after correction, it is clear to see that the regions belonging to the vehicle (in yellow box) become “brighter” after correction. This is because after correction, the reconstructed 3D points located on the surface of the vehicle become more vertical to the ground.

### 6.2. Occupancy and Motion Indicator

The corrected 3D points are assigned to cells with pre-defined sizes with respect to their positions. In each cell *C*(*i, j*), the number of assigned points *n_i,j_* is counted. In addition, the number of 3D points extracted from the independent moving segmentation calculated in Section 5.2 is counted as $n_{i,j}^{d}$. When trying to compute a cell's occupancy probability with respect to the number of 3D points it holds, a similar problem is encountered again as in Section 5.2. Looking at Figure 7a, the cells on the ground near the stereoscopic system always hold more points than the grids far away. Directly estimating the occupancy probability from the absolute number of points would always lead to a faulty decision. In the literature, [2,24] do not mention this problem, while [25,26] avoid it by a previous separation of road pixels. In our method, this problem is overcome also by sigmoid function-based adjustment. The absolute number of points for cell *C*(*i,j*) is adjusted as:

(9) $$n_{i,j}^{\prime }=n_{i,j}\cdot \text{S}(d_{i,j})=n_{i,j}\cdot \frac{r}{1+e^{d_{i,j}*c}}$$

where *n_i,j_* and $n_{i,j}^{\prime }$ are the absolute and adjusted number of points in cell *C*(*i,j*), respectively. *d_i,j_* is the distance from the stereo vision system to the grid. *r* and *c* are control coefficients. The occupancy probability with respect to number of points is modeled as:

(10) $$P_{i,j}(O|\text{num})=1-e^{-(n_{i,j}^{\prime }/\delta_{n})}$$

where *δ_n_* is a scale factor. Equation (10) means that the greater the number of points in a cell, the greater the probability of it being occupied. This probability model is similar to [26]. However, it is not convenient to directly use the probability in decision-making. The log-odds of the probability are then adopted:

(11) $$l_{i,j}(O|\text{num})=\log(\frac{P_{i,j}(O|\text{num})}{1-P_{i,j}(O|\text{num})})$$

The average height *h̄_i,j_* of 3D points in a cell *C*(*i,j*) could be helpful when an obstacle is not perpendicular to the ground. The probability and corresponding log-odds are similar to Equations (10) and (11).

(12) $$P_{i,j}(O|\text{height})=1-e^{-({\bar{h}}_{i,j}/\delta_{\bar{h}})}$$

(13) $$l(O|\text{height})=\log(\frac{P(O|\bar{h})}{1-P(O|\bar{h})})$$

where *δ_h̄_* is a scale factor. Similarly, Equation (12) means that the higher the average height of points in a cell, the greater the probability of it being occupied. The final log-odds of occupancy for a cell are set as a weighted average of the two log-odds in Equations (11) and (13):

(14) $$l_{i,j}(O)=w_{n}l_{i,j}(O|\text{num})+w_{\bar{h}}l_{i,j}(O|\text{height})$$

where *w_n_* and *w_h̄_* are the weights with *w_n_ + w_h̄_* = 1. Based on the two log-odds of each cell *C*(*i, j*), the occupancy indicator *O_i,j_* is decided as:

(15) $$O_{i,j}=\{\begin{array}{ll} \text{undetected} & \text{if}\;n_{i,j}^{\prime }<n_{t}\\ \text{occupied} & \text{if}\;l_{i,j}(O)\geq l_{t}\\ \text{free} & \text{if}\;l_{i,j}(O)<l_{t} \end{array}$$

where *n_t_* and *l_t_* are thresholds manually set for making a decision.

For the motion indicator *M_i,j_*, this is decided by comparing $n_{i,j}^{d}$, the number of 3D points from independent moving segmentations, with $n_{i,j}^{s}$, the number of 3D points from the static image region.

(16) $$M_{i,j}=\{\begin{array}{ll} \text{dynamic} & \text{if}\;n_{i,j}^{d}>n_{i,j}^{s}\\ \text{static} & \text{otherwise} \end{array}$$

## 7. Experiments in a Real Environment

### 7.1. Implementation

The proposed framework is evaluated by our experimental vehicle, SetCar (Figure 2), introduced in Section 3.1. The installed stereo vision system (Bumblebee XB3) observes the surroundings by stereo image pairs (with a resolution of 640 × 480) in a frame-rate of 10 fps with baseline length of 0.24 m. The whole framework is implemented in C++, based on the OpenCV library http://opencv.org/, without any acceleration technique. A desktop computer with a CPU Intel i7-3770 quad core 3.40 GHz running Linux is used to run the software. We will firstly discuss the performances of sparse feature point-based motion analysis and, then, evaluate the dynamic grid mapping results. The data set contains more than 4000 images acquired by our SetCar platform when driving in the city of Belfort, France.

### 7.2. Evaluating Sparse Feature Point-Based Motion Estimation

In Sections 5.1.1 and 5.1.2, we have reviewed several image feature detectors and descriptors. Here, we will evaluate their performances for motion analysis in our application. The evaluated feature detectors are: GFTT, FAST, SIFT, SURF, ORB, STAR and BRISK; and the feature descriptors are: SIFT, SURF, ORB and BRISK. For a fair comparison, all of the feature detectors and descriptors use default parameters implemented in OpenCV.

#### 7.2.1. Experiments in Feature Detectors

Several general criteria used for evaluating the feature detector are given in [38,45]. Being different from applications, such as object recognition, which usually have prominent scale or viewpoint changes, in our applications, the detectors are used to establish point correspondences between consecutive video frames. Therefore, the scale and viewpoint changes are too small to be evaluated in our application. The performances of feature point detectors in our application are evaluated from 3 aspects: repeatability, uniformity and speed.

- Repeatability: Given two images of the same scene acquired in different conditions, the percentage of features that could be found in both images (the reference image and the image after transformation) is defined as repeatability. The repeatability score is defined as: *S_r_* = *f*^−^/f*, where *f*^−^ is the number of features found in both images and f* is the number of features detected in the reference image.
- Uniformity: To precisely estimate motion in dynamic scenes, the detected features should be distributed as uniformly as possible. To evaluate uniformity, we divide the 640 × 480 image into *n_u_* disjoint identical cells. If the number of feature points located in one grid reaches is more than 2, it would be marked as “filled”. The uniformity score is: $S_{u}=n_{u}^{-}/n_{u}$, where $n_{u}^{-}$ is the number of “filled” cells.

**Figure 8.**
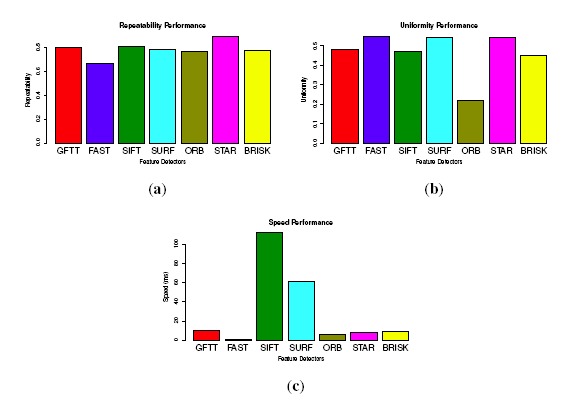
Performances of the feature detectors. (a) Repeatability performance; (b) uniformity performance; (c) speed performance.

Ten typical and different images from the database are selected for testing the feature detectors. To evaluate repeatability, we use a similar method as [45], by converting an image by a homography transformation. The homography provides a ground truth to find corresponding features in new image. For the uniformity, the original image is divided into 192 cells with a size of 40 × 40. The repeatability and uniformity are calculated as stated above. The performances are shown in Figure 8. All of the tested values are the average of the samples. For the repeatability, STAR performs the best, attaining 0.9, followed by GFTT (0.8), and FAST performs the worst (0.64). For the uniformity, FAST performs the best with a uniformity score of 0.67, followed by STAR, SURF, GFTT and SIFT, and ORB performs the worst. While for the speed, FAST is much more faster than the other detectors, GFTT, ORB and STAR are almost 10-times slower than FAST, and SIFT is more than 100-times slower than FAST.

#### 7.2.2. Experiments in Establishing Point-to-Point Correspondences

To evaluate the process of building point-to-point correspondences through four images, two approaches (KLT tracker-based and feature descriptor matching-based) are considered. Since feature descriptors can be applied in most kinds of detectors, we consider only original detector-descriptor combinations: “SIFT detector + SIFT descriptor”, “SURF + SURF descriptor”, “ORB detector + ORB descriptor” and “BIRSK detector + BRISK descriptor”. The nearest neighbor matching method [35] is used when matching feature descriptors. The KLT tracker is used for the detectors of GFTT, FAST and STAR. To evaluate performances, we propose two criteria:

- Survival rate: Both the KLT tracker and feature matching method would lose a part of the feature points because of inaccurate tracking or matching. When tracking or matching through four images, this phenomenon could be amplified and cause severe problems. The survival rate is calculated by $\text{survivalrate}=\frac{n_{\text{left}}}{n_{\text{whole}}}$, where *n_whole_* is the number of whole detected features and *n_left_* is the number of successfully tracked or matched features.
- Accuracy: The accuracy of established point-to-point correspondences can be evaluated from the ratio between inliers and the whole features when applying RANSAC for ego-motion estimation. The accuracy factor is: $\frac{n_{\text{inlier}}}{n_{\text{whole}}}$. To exclude distractions of independent moving objects, we only use images without moving objects.

**Figure 9.**
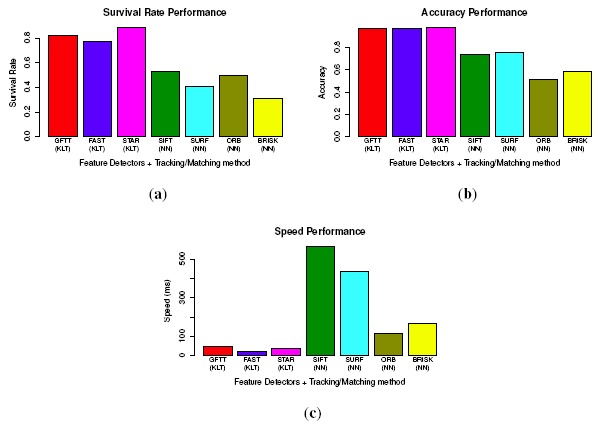
Evaluating the process of circular building point-to-point correspondences. (**a**) Survival rate; (**b**) accuracy factor; (**c**) speed performance.

Forty images (10 groups of consecutive stereo image pairs) are selected from the image database. For a fair comparison, we control the number of detected feature points between 400–500. The evaluation results of survival rate, accuracy, as well as speed are shown in Figure 9. In the survival rate, STAR achieves the best performance, and BRISK gets the worst. Moreover, all of the KLT feature tracking-based methods perform better than the nearest neighbor feature matching method. This is because the KLT tracker-based method only detects features once, while matching-based methods have to detect feature points in all four images. The repeatability evaluated in Figure 8 significantly reduces the number of finally matched feature points. For accuracy, all of the KLT tracker-based methods perform better than the feature matching-based methods again. This means that the noises in the KLT tracker-based methods are less. This could be explained by the merit of corner-like features in terms of accurate localization in images. As for the speed, although binary descriptors prominently reduce the matching time, KLT tracker-based methods still perform much better than all of the matching-based methods. In fact, the feature descriptors are usually applied in fields, such as object recognition or image stitching or registration, where the changes between different images are big and complex (scale, affine transform). In such situations, the KLT tracker will fail. However, in our application of small image changes, the KLT tracker is proven to perform the best. Therefore, we choose the STAR feature detector and the KLT tracker to establish point correspondences.

### 7.3. Experiments in Dynamic Occupancy Grid Mapping

The region of interest (ROI) for the grid map is set to 20 m × 20 m, with a maximum height of 3 m. The parameters used to calculate the occupancy indicator are set as: *β* = 0.01, *δ_n_* = 0.2, *δ_h̄_*, *w_n_* = *w_h̄_* = 0.5, *n_t_* = 1.5, *l_t_* = 7, *r* = 8, *c* = 0.02 (all of the parameters are tuned manually). The resolution of the map is set to 200 × 200; hence, the size of each cell is 10 cm × 10 cm

**Figure 10.**
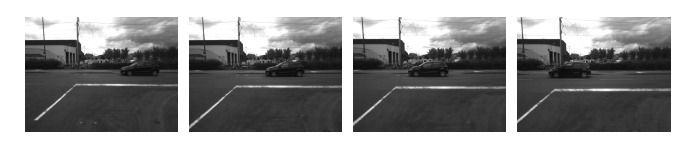

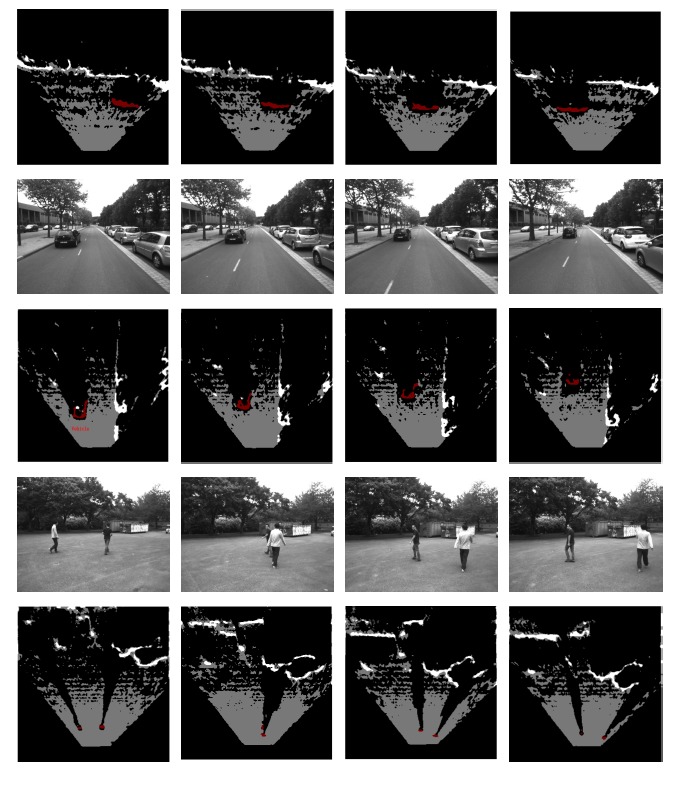
Experimental results of the dynamic occupancy grid map: independent moving objects (red), static occluded areas (white), free areas (gray) and undetected areas (black).

**Figure 11.**
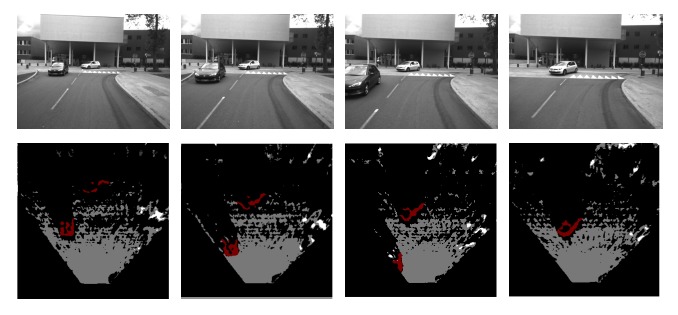
More experimental results of the dynamic occupancy grid map: independent moving objects (red), static occluded areas (white), free areas (gray) and undetected areas (black).

Figures 10 and 11 show the performances of the proposed method in 4 typical video sequences. The corresponding dynamic occupancy grid mapping results are shown under the sequences. Dynamic occupied areas are labeled in red, the white and gray cells represent occupied static areas and free space, respectively, while black cells are undetected areas. The Table 1 represents the precision evaluation of the proposed independent moving objects segmentation method in the 4 sequences. “TP” and “FP” are short for “true positive” (detecting and segment moving objects successfully) and “false positive” (taking static objects as dynamic objects; this is usually caused by noisy matched feature points). From the results, we could see that the proposed framework performs well when there are moving vehicles in the scenario. However, the performance degenerates when pedestrians appear. The major reason is that a moving pedestrian is slower than a moving vehicle. Hence, several detected feature points in a moving pedestrian are classified as static. This problem would cause a moving pedestrian to not be detected. At last, the whole computation time including motion analysis and dynamic occupancy grid mapping is 0.5 s on average (SGBM disparity calculation (0̃.25 s), visual odometry (0̃.1 s), moving object detection and segmentation (0̃.1 s), dynamic occupancy grid mapping (0̃.05 s)).

## 8. Conclusions and Future Works

In this paper, we present a framework of a dynamic occupancy grid mapping technique. The framework mainly consists of motion analyzing and dynamic occupancy mapping. The motion estimation is achieved based on circularly-tracked feature points. A U-V disparity map-based independent moving object segmentation is also presented. The dynamic occupancy grid mapping is performed with a 3D reconstructed point cloud. The combination of the segmentation of a moving object and the occupancy probability estimation results in the final dynamic occupancy grid map. For future works, we are planning to improve the performance when many pedestrians appear, to make dynamic occupancy grid mapping smoother and to integrate LiDAR measurements within the data fusion framework.

## Figures and Tables

**Table 1.** Quantitative analysis in 4 sequences acquired by our platform.

	**True Positive**	**False Positive**	**Sequence Length**
Sequence 1	97.5%	2.0%	221
Sequence 2	95.5%	3.5%	235
Sequence 3	93.4%	4.4%	217
Sequence 4	89.4%	9.6%	202
